# The striatum, the hippocampus, and short-term memory binding: Volumetric analysis of the subcortical grey matter's role in mild cognitive impairment

**DOI:** 10.1016/j.nicl.2019.102158

**Published:** 2019-12-29

**Authors:** Maria C. Valdés Hernández, Rupert Clark, Szu-Han Wang, Federica Guazzo, Clara Calia, Vivek Pattan, John Starr, Sergio Della Sala, Mario Alfredo Parra

**Affiliations:** a College of Medicine and Veterinary Medicine, University of Edinburgh, Edinburgh, United Kingdom; b Centre for Clinical Brain Sciences, University of Edinburgh, Edinburgh, United Kingdom; c Dementia Research Institute, University of Edinburgh, Edinburgh, United Kingdom; d Row Fogo Centre for Research into Ageing and the Brain, University of Edinburgh, Edinburgh, United Kingdom; e Human Cognitive Neuroscience, Psychology, University of Edinburgh, Edinburgh, United Kingdom; f School of Health in Social Sciences, University of Edinburgh, Edinburgh, United Kingdom; g Stirling Community Hospital, Stirling, United Kingdom; h Alzheimer Scotland Dementia Research Centre, University of Edinburgh, Edinburgh, United Kingdom; i School of Psychological Sciences and Health, University of Strathclyde, Glasgow, United Kingdom; j Department of Psychology, Universidad Autónoma del Caribe, Barranquilla, Colombia

**Keywords:** Basal ganglia, MRI, Ageing, Cognition, Memory binding, Hippocampus, Globus pallidus

## Abstract

**Background:**

Deficits in short-term memory (STM) binding are a distinguishing feature of preclinical stages leading to Alzheimer's disease (AD). However, the neuroanatomical correlates of conjunctive STM binding are largely unexplored. Here we examine the possible association between the volumes of hippocampi, parahippocampal gyri, and grey matter within the subcortical structures – all found to have foci that seemingly correlate with basic daily living activities in AD patients - with cognitive tests related to conjunctive STM binding.

**Materials and methods:**

Hippocampal, thalamic, parahippocampal and corpus striatum volumes were semi-automatically quantified in brain magnetic resonance images from 25 cognitively normal people and 21 patients with Mild Cognitive Impairment (MCI) at high risk of AD progression, who undertook a battery of cognitive tests and the short-term memory binding test. Associations were assessed using linear regression models and group differences were assessed using the Mann-Whitney U test.

**Results:**

Hippocampal and parahippocampal gyrus volumes differed between MCI and control groups. Although the grey matter volume in the globus pallidus (*r* = -0.71, *p* < 0.001) and parahippocampal gyry (*r* = -0.63, *p* < 0.05) correlated with a STM binding task in the MCI group, only the former remained associated with STM binding deficits in MCI patients, after correcting for age, gender and years of education (β = -0.56,*P* = 0.042) although with borderline significance.

**Conclusions:**

Loss of hippocampal volume plays no role in the processing of STM binding. Structures within the basal ganglia, namely the globus pallidus, could be part of the extrahippocampal network supporting binding. Replication of this study in large samples is now needed.

## Highlights

• Hippocampal atrophy plays no role in short-term memory binding.
• The globus pallidus could be part of the brain network supporting binding.
• Total brain atrophy does not correlate with striatal grey matter atrophy in MCI.
• Striatal grey matter atrophy reflects in total brain atrophy in controls.
• Hippocampal and parahippocampal volumes correlate in MCI and controls.

## 1 Introduction

As people reach older age, cognitive deterioration is paralleled by the atrophy of multiple brain regions, distinctively seen in dementias such as Alzheimer's disease (AD) (Dubois et al., 2016; Petersen, 2004; Petersen et al., 2009). Within the continuum of cognitive decline with advanced age leading to AD, mild cognitive impairment (MCI) is considered a stepping stone that confers a massively increased risk of progression to AD in diagnosed individuals (Korolev et al., 2016). However, only a fraction of individuals with MCI are known to progress to AD (Lazarczyk et al., 2012). amidst the tests that show promise in predicting progression to AD stands out a cognitive test that assesses short-term memory (STM) binding (Parra et al., 2010; 2011; Della Sala et al., 2016; see Costa et al., 2017 for recent consensus recommendations), once it has been discovered that deficits in the ability to temporarily hold bound features such as shapes and colours (i.e., conjunctive binding) are present in the pre-clinical stages of AD (Parra et al., 2010; 2011). Studies have found that whilst patients in these stages of familial AD showed significantly affected conjunctive STM binding functions, their memory for associative information (e.g., word pairs) also known as relational long-term memory (LTM) binding was unimpaired (Parra et al., 2010; 2011). Furthering this, it has been shown that STM binding is resilient to neurological changes brought about by depression (Parra et al., 2010), and that it could distinguish AD patients from other forms of dementia (Della Sala et al., 2012; Cecchini et al., 2017). It has also been found that normal ageing has no significant impact on STM binding, despite a decline in overall visual STM capacity (Brockmole and Logie, 2013; Hoefeijzers et al., 2017; Parra et al., 2009; Rhodes et al., 2017).

Research on memory binding functions in normal individuals and clinical populations has grown considerably over the last decade. Relevant to this study are two forms of memory binding, namely relational and conjunctive. Both forms of binding were described in the long-term memory (LTM) related literature (Mayes et al., 2007; Moses and Ryan, 2006; Olsen et al., 2012) emphasising that whereas relational binding was clearly reliant on the functional integrity of the hippocampus, less was known about the latter (i.e. conjunctive memory binding), particularly in STM (Moses and Ryan, 2006). In LTM, conjunctive binding seems to support the formation of structural representations through repetition, a slow learning process which involves interaction between the hippocampus and the neocortex. Relational binding supports the formation of associative representations between items which hold different identities. This type of binding is considered a building block of episodic memory (Tulving, 2002) as it allows linking *what, where*, and *when* in LTM. Contrary to the flexible nature of relational representations, which can be accessed and retrieved either via a constituent element or a whole episode and altering the association between elements leaving their identities intact, conjunctive representations are rigid as modifying any aspect of such memories will lead to new representations or objects’ identity. Relevant to the present study is a form of conjunctive binding in STM which supports the integration of objects’ feature within unified representation. The choice of this particular binding task for the purpose of the current study is that it is not affected in healthy ageing (Brockmole et al., 2008), though it is impaired in early AD (Parra et al., 2009). Moreover, it is not affected by repeated testing (Logie et al., 2009), or by the level of education (Parra et al., 2011), so it can be used to test people with low levels of literacy as well as people who are highly educated and in assessing patients with very different socio-cultural backgrounds (Parra et al., 2011).

As pointed out by (Moses and Ryan, 2006), knowledge on the neuroanatomical correlates of conjunctive binding in STM is still in its infancy and, as such, the circuitry involved is largely unknown. Targeted structures include the hippocampus, which traditionally is seen as an essential component of LTM function and has been repeatedly linked to relational memory binding functions regardless of the memory system (i.e. LTM or STM); (Liang et al., 2016; Parra et al., 2015) (Parra et al., 2019). However, several studies involving hippocampal lesions have shown that conjunctive STM binding is preserved despite hippocampal atrophy (Baddeley et al., 2011; Parra et al., 2019; Parra et al., 2015; Zokaei et al., 2018). A fMRI study by Parra et al. (2014) looking at regional neural correlates of a conjunctive STM binding task found no significant activation of the hippocampus or medial temporal lobe. Another study by Parra et al. (2015) looking at a stroke patient with a lesioned right hippocampus found that conjunctive STM binding was completely unimpaired, despite a relational STM binding deficit. More recently, Jonin et al. (2019) confirmed a selective impairment of relational binding when the hippocampal system is compromised as shown by clinically relevant memory tests in a patient with developmental amnesia due to bilateral damage to the hippocampus at birth (see Baddeley et al., 2010 for a similar finding). The latter study is of particular relevance as the authors contrasted tests of relational LTM commonly used in clinical settings, which are known to assess associate memory functions of the hippocampus (e.g., PAL-CANTAB, Four-Mountain Test, FCRST) with tests of conjunctive STM binding. The patient who features in this case study failed all the relational memory tests but succeeded on all the conjunctive memory tests. These studies bolster a growing body of work that challenges the notion that hippocampi are the key neurological loci for relational but not conjunctive STM binding (Parra et al., 2019; Parra et al., 2015). Furthermore, they also challenge the notion that the hippocampus and its associated functions should be the target of cognitive assessments aimed at the early detection of AD (Dubois et al., 2010; Dubois et al., 2016a, Dubois et al., 2016b). Although the evidence discussed above, gathered from cognitively normal individuals and patients with neurological disorders that are not part of the continuum of cognitive decline of progression to dementia, lends support to the hypothesis of a hippocampal-independent conjunctive STM binding function, such a hypothesis has never been addressed in patients with neuroprogressive diseases that cause dementia. One possibility is that in such individuals the hippocampus would support aspects of STM processing needed to temporarily retain feature bindings (see for example Yonelinas, 2013). A second possibility is that cortical areas involved in components of the STM network supports STM binding in these patients. For instance, frontal and parietal-occipital cortices have been already reported as correlates of conjunctive STM binding in patients with sporadic or familial MCI (Parra et al., 2017; Pietto et al., 2016). A third, underexplored, possibility is that striatal and/or thalamic grey matter support STM binding in these patients.

Striatal structures have been shown to associate with working memory (Eriksson et al., 2015). A review of the literature up to 2016 on brain atrophy in AD and ageing highlights that few studies have reported significant atrophy in striatal structures in AD (Pini et al., 2016). It concludes that the impact of these changes in cognition still remains unclear; and hypothesises that, in AD, atrophy in these structures might be secondary to degeneration of structures connected to them as the hippocampus (Pini et al., 2016). The timeline of these structural changes in the continuum of cognitive decline to dementia is still unknown. A voxel-based morphometry analysis revealed small striatal clusters to be correlated with basic daily living activities in patients with AD on a study that mapped the neuroanatomy of functional decline in AD from basic to advanced activities of daily living (Slachevsky et al., 2019). Moreover, amyloid signal in the striatum improves the description of AD progression (Hanseeuw et al., 2018). However, the striatal input in STM binding remains unexplored.

Similarly, in recent years, evidence from multiple studies support novel views of thalamic functions that emphasize integrative roles in cognition, including memory (Bradfield et al., 2013a; Wolff et al., 2015; Alcaraz et al., 2016; Cholvin et al., 2013; Mitchell and Dalrymple-Alford, 2006), especially due to its role in subcortical integration (Mitchell et al., 2002), key for maintaining and updating representations (Loureiro et al., 2012; Sweeney-Reed et al., 2014). A recent study proposes that the large range of diverse and apparently separate cognitive functions that have been associated to the thalamus, ranging from learning and memory to flexible adaptation, may indeed be supported by a more general role in shaping mental representations (Wolff and Vann, 2019). Several features of thalamocortical circuits are consistent with this role, suggesting that divergent and convergent thalamocortical and corticothalamic pathways may complement each other to support these functions. Its association with STM binding is, however, unknown, despite suggestions on its possible involvement in memory binding tasks (Bradfield et al., 2013b; Wolff and Vann, 2019).

This study aims to first establish whether the conjunctive STM binding task differentiates individuals with and without MCI and then identify the key subcortical and whole-brain correlates for this function. The well-established hippocampal and parahippocampal (i.e. entorhinal) atrophy in AD (Dubois et al., 2014; Amirnoff, Kveraga and Bar 2013; Arlt et al., 2013; Pini et al., 2016) fully justifies the volumetric examination of these medial temporal lobe regions in addition to that from the rest of the subcortical grey matter areas, which have been largely unexplored in relation to memory binding, despite indications of their possible involvement. Two memory loads were used for the STM binding tasks and the baseline STM task (i.e., single feature such as shape only). Traditional neuropsychological tests were also used to in the assessment such as the Addenbrooke's Cognitive Examination (ACE), Trail Making Task (TMT B-A), Hopkins Verbal Learning Tasks (HVLT) and verbal fluency tasks (Total FAS and Animal Fluency). Processing speed was assessed by a Digit Symbol Substitution task and Rey Figure drawing tasks and the language ability was assessed by a Graded Naming Test. By analysing the volume of grey matter (GM) within the striatal structures, thalami and hippocampi from brain magnetic resonance imaging (MRI), in cognitively normal and MCI patients who undertook a baseline STM and conjunctive STM binding tasks with two different memory loads, we hope to shed further light on the striatal and hippocampal involvement in STM and conjunctive STM binding. Given that general atrophy is a sensitive marker of cognition in the elderly (Royle et al., 2013), and one of the main signatures in AD (Pini et al., 2016), we also want to explore to which extent general atrophy (i.e. given by total brain tissue and normal-appearing white matter volumes) is involved in the conjunctive STM binding functions evaluated here. We hypothesise that the volume of GM in the basal ganglia structures, and possibly in the thalami,could be associated with the performance on STM binding tasks.

## 2 Materials and methods

### 2.1 Study participants

The sample used in this study was composed of 26 cognitively normal individuals and 24 non-amnestic patients diagnosed with MCI matched for age and years of education, which consented to participate in a study of cognitive ageing (Parra et al., 2016) funded by the UK Alzheimer's Society (https://www.alzheimers.org.uk/research/our-research/research-projects/can-identifying-problems-short-term-memory-help-diagnose-alzheimers-disease). Patients under the care of a geriatrician (JS) and an old age psychiatrist (VP), were referred to the primary study that provided the data after confirming they met inclusion criteria (aged 60+, changes in memory, or outcome of relevant neuropsychological tests carried out as part of their routine examination, due to concerns on thinking abilities reported by patients or close relative, unlikely explained by cerebrovascular disease or depression, and not sufficient to meet criteria for dementia). Control participants reported no health problems or significant clinical history. They were recruited through the panel of volunteers of the Psychology Department of the University of Edinburgh who also applies a battery of neuropsychological tests as part of their recruitment procedure (https://www.ed.ac.uk/ppls/psychology/research/volunteering). All participants gave their informed consent to take part in the study. The primary study that provided data for this study was approved by the relevant NHS Scotland Research Ethics Committees (Ref: 06/MRE07/40). We used all structural imaging, demographic and cognitive data available.

### 2.2 MRI acquisition

Structural MRI was performed on a GE Signa Horizon HDx 1.5 Tesla MRI scanner following the imaging acquisition protocol from a large study on cognitive ageing (Wardlaw et al., 2011). Sequences acquired were: T1-, T2-, T2*- and Fluid Attenuated Inversion Recovery (FLAIR)-weighted whole brain scans. Sequence parameters are described in Table S1 of the Supplementary material.

### 2.3 Image analysis

**Fig. 1:**
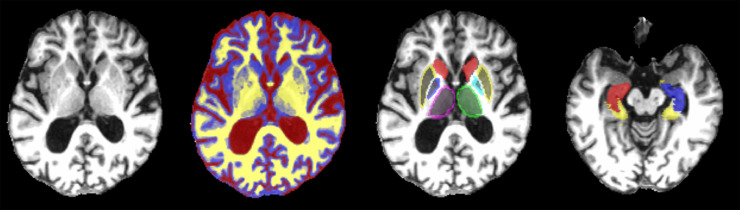
T1-weighted Magnetic Resonance Imaging axial slices of a study participant with the tissue and ROI segmentation results superimposed in different colours. From left to right: brain extracted T1-weighted axial slice; the same brain slice with the cerebrospinal fluid represented in red, the grey matter in blue and the white matter in yellow; the same slice with the thalami (magenta and green), globus pallidi (cyan and blue), putamen (yellow) and caudate (red); other axial slice showing the hippocampi (red and blue) and parahippocampal gyri (yellow).

Contents of intracranial volume (ICV) within the inner skull table including normal appearing white matter, cerebrospinal fluid, brain tissue, veins and dura, were initially extracted automatically using the brain extraction tool (BET2) from the FMRIB software library (FSL) (https://fsl.fmrib.ox.ac.uk/fsl/fslwiki). Binary ICV masks were visually checked and manually edited in axial, coronal and sagittal planes when required using Mango (http://ric.uthscsa.edu/mango/). Normal appearing white matter (NAWM) and cerebrospinal fluid (CSF) were segmented using another tool from the same library: the FSL Automatic Segmentation Tool (FAST) with the default settings and the brain extracted T1-weighted image as input. This tool uses a hidden Markov random field model and an associated Expectation-Maximization algorithm to produce a 3D volume where each voxel has values between 0 and 1 indicating the probability of it being CSF, grey matter or NAWM (Zhang et al., 2001). The structures of the basal ganglia, hippocampi and thalami were extracted using a combination of other three tools from the same library: Smallest Univalue Segment Assimilating Nucleus (SUSAN) to reduce noise, FMRIB's Linear Image Registration Tool (FLIRT) and a model-based segmentation/registration tool (FIRST), combined on an automatic pipeline developed in-house described in details previously (Valdés Hernández et al., 2015). This result was visually assessed for further manual editing when it was required. The probabilistic maps resultant from the tissue segmentation were combined with the results from the subcortical segmentations using partial volume estimates to discern the volume of the GM tissue in the subcortical structures. Parahippocampal gyrus volumes were assessed using T1W sequences and edited manually using Mango in axial, coronal and sagittal planes. All brain volumes were adjusted for head size related differences using ICV. All measurements were performed blinded to any other clinical, cognitive or demographic information. Two observers (RC and MVH) assessed each segmentation result, and discrepancies were discussed until a final agreement was reached. Fig. 1 illustrates an example of the segmentation results.

To explore voxel-wise differences between the regions of interest (ROIs) segmented in both groups, we mapped all participants’ ROIs to an age-relevant (76 years old) template (https://datashare.is.ed.ac.uk/handle/10283/1957) (Dickie et al., 2016) and generated, separately, the probability distribution map of these ROIs in controls and MCI patients.

### 2.4 Cognitive assessments

All participants undertook a comprehensive neuropsychological interview prior to or soon after the MRI scan (± up to 1 month apart). Pre-morbid intelligence was assessed using the Test of Premorbid Function (TOPF; Wechsler), and a perceptual binding task was used to screen participants in order to assess whether they were capable of forming and processing bindings in perception (Parra et al., 2010). Following initial screening, Addenbrooke's Cognitive Examination (ACE-R) (Mioshi et al., 2006) was performed to further assess neuropsychological impairments, and executive functions were assessed using the difference between Trail Making Task (TMT B-A), and verbal fluency tasks (COWAT - letter and animal naming fluency, Sumerall et al., 1997). Hopkins Verbal Learning Tasks (HVLT) (Benedict et al., 1998) for recognition, delayed recall and total recall were used to assess memory and learning of word lists. Processing speed was also assessed using a Digit Symbol Substitution task (Wechsler, 1997), and Copy and Recall of Rey Figure (Osterrieth, 1944) drawing tasks were used to assess visual immediate and delayed recall ability. A Graded Naming Test (McKenna and Warrington, 1980) was used to provide a language ability score (Parra et al., 2010).

Conjunctive STM binding was assessed using the procedures set out by Parra et al. (2010). The STM tasks presented three conditions, one assessed memory for Shape Only and two assessed memory for Shape-Colour Binding. The former condition served as the baseline. Two memory loads were used in the assessment. The baseline condition and one condition assessing STM binding presented 3 items. We included an additional condition to assess STM binding at low memory load (2 items). We have recently noted that STM tasks assessing conjunctive binding should consider memory load with the regard to study's hypothesis (e.g., whether the cost of feature binding in STM is relevant) and the studied population (i.e., clinical samples with different disease severities). We decided to subject such an issue to further investigation within the neuroanatomical context. Typically, the STM binding test asks participants to identify changes occurring across two consecutives displays, a study and a test display. Changes can involve new features replacing features previously studied as it is the case for the Shape Only condition, or studied features swapping between items during the test display, as it happens in the Shape-Colour Binding condition. To detect the former, memory for individual features suffices whereas for the latter, memory for the binding between features is additionally needed. We recorded the accuracy with which participants recognised changes or sameness.

### 2.5 Statistical analyses

Statistical analyses were performed using IBM SPSS Statistics 21 and double-checked using MATLAB R2014a (scripts provided as supplementary material for reproducibility and comparability purposes). Due to the small sample size, and to avoid overestimation in the significance of our results, all tests were repeated using bootstrap with *n* = 1000 samples. Group comparison was done using the Independent Samples T-Test for equality of means with bootstrap. The Levene's test for equality of variances showed that variances were unequal for all cognitive and imaging variables. We performed a two-tailed bivariate Pearson's cross-correlation between all brain volumetric and cognitive variables used to explore: 1) whether the bivariate relations between these variables followed a distinct pattern in each group, 2) whether the covariates used in the regression models (i.e. age, biological sex and years of education) were related with any of the cognitive and imaging variables in the sample, and 3) the bivariate relationships between imaging and cognitive variables in the whole sample. Full bootstrapped results are given in supplementary data spreadsheets considering both pair-wise and list-wise exclusion of missing values, for transparency. All results were corrected for multiple comparisons using false discovery rate (FDR). The p-values and their correspondent q-values containing measures of hypothesis testing error for each observation, resultant from this analysis (Storey, 2002) are also given in a supplementary data spreadsheet. We performed linear regression using general linear models that considered only main effects to investigate possible associations between brain volumetric variables (i.e. independent variables) and cognitive task performance (i.e., dependent variables) within control and MCI groups (i.e., selection variable) when accounting for age, gender and years of education (i.e., covariates). The percentage of CSF volume in ICV was used as a surrogate for total brain atrophy in all analyses. The type one error was set at *P* < 0.05.

## 3 Results

### 3.1 Sample characteristics

The descriptive statistics of the sample are given in Table 1. More details (including the descriptive stats on age, gender, and measurements) can be found in the supplementary Table S2 and graphically in supplementary Figures S1 and S2. Our final sample consisted of 21 MCI patients and 25 control participants of comparable age and education, who had both adequate MRI sequence data to assess the imaging parameters involved in the analyses (e.g. normal tissues, GM in subcortical structures and parahippocampal gyrus), and completed the cognitive tests. The discrepancy with the initial recruitment figures were lack of relevant structural images due to incomplete scan sessions (2 cases), and/or absence of cognitive data (4 cases).

**Table 1:** Descriptive statistics of the sample (mean ± SD) (*n* = 46).

		Control Group (*n* = 25)	MCI Group (*n* = 21)
**Demographic Variables (years)**	Men/Women	8/17	13/8
	Age	76.24 ± 5.37	74 ± 5.49
	Education	15.08 ± 3.58	13.57 ± 3.88
**Cognitive Assessment Scores**	STM Shape-Only 3	0.89 ± 0.07	0.84 ± 0.08
	STM Shape-Colour Binding 3	0.71 ± 0.10	0.63 ± 0.10
	STM Shape-Colour Binding 2	0.91 ± 0.11	0.81 ± 0.13
	ACE	94.42 ± 5.42	84.33 ± 7.95
	TMT B-A	56.96 ± 42.60	78.19 ± 35.32
	HVLT Recognition	10.25 ± 2.19	8.70 ± 2.41
	HVLT Delayed Recall	7.32 ± 3.68	3.90 ± 3.62
	HVLT Total Recall	24.64 ± 6.34	17.24 ± 5.17
	Total FAS	47.48 ± 12.51	32.52 ± 14.52
	Animal Fluency	19.72 ± 4.26	10.90 ± 5.33
	Digit Symbol	55.96 ± 13.54	42.86 ± 9.42
	Rey Figure Copy	31.66 ± 7.39	31.52 ± 3.77
	Rey Figure Immediate Recall	18.29 ± 7.85	11.57 ± 9.02
	Rey Figure Delayed Recall	17.87 ± 6.81	12.72 ± 8.80
	Graded Naming Test	23.00 ± 4.06	19.24 ± 3.99
**Imaging Variables (% in ICV)**	Normal Appearing White Matter	32.68 ± 1.52	32.69 ± 1.24
	Cerebrospinal Fluid	30.76 ± 2.50	31.45 ± 1.66
	Left Hippocampus	0.239 ± 0.046	0.214 ± 0.050
	Right Hippocampus	0.245 ± 0.040	0.209 ± 0.049
	Total Hippocampus	0.484 ± 0.078	0.423 ± 0.094
	Left Caudate Nucleus	0.192 ± 0.043	0.181 ± 0.047
	Right Caudate Nucleus	0.207 ± 0.033	0.180 ± 0.052
	Left Putamen	0.235 ± 0.050	0.216 ± 0.049
	Right Putamen	0.239 ± 0.050	0.226 ± 0.049
	Left Globus Pallidus	0.020 ± 0.008	0.014 ± 0.008
	Right Globus Pallidus	0.019 ± 0.010	0.012 ± 0.008
	Left Thalamus	0.238 ± 0.044	0.245 ± 0.048
	Right Thalamus	0.231 ± 0.041	0.236 ± 0.052
	Parahippocampal Gyrus	0.279 ± 0.114	0.218 ± 0.050

Legend: VSTM: Visual Short Term Memory, ACE: Addenbrooke's Cognitive Examination, TMT B-A: Trail Making Task B-A, HVLT: Hopkins Verbal Learning Task.

### 3.2 Group comparison

#### 3.2.1 Group comparison between cognitive variables

Group comparison of cognitive performance outcomes yielded highly significantly lower mean values of STM binding with load 2 (see task description above) ACE, HVLT total recall, and total FAS, animal fluency, digit symbol, Rey figure immediate recall and graded naming test for the MCI group compared to the controls (*P* = 0.021 – 0.001, Table 2, Supplementary Figure S1). Mean group differences in the outcome of the STM task presented Shape-Only (with 3 objects), HVLT delayed recall and Rey figure delayed recall had only marginal significance (*P* = 0.041 - 0.043).

**Table 2:** Results of the Independent samples T-test (based on 1000 bootstrap samples, equal variances not assumed).

Parameter	Mean difference [95% Confidence Interval]	Std. Error	p-value
**STM Shape-Only 3**	0.058 [0.002 0.107]	0.026	**0.043**
STM Shape-Colour Binding 3	0.058 [−0.011 0.134]	0.035	0.112
**STM Shape-Colour Binding 2**	0.106 [0.023 0.192]	0.043	**0.021**
**Addenbrooke's Cognitive Examination (ACE)**	10.762 [5.785 16.196]	2.605	**0.003**
Trail Making Task B-A	−20.879 [−47.441 7.351]	13.723	0.155
Hopkins Verbal Learning Task recognition	1.381 [−0.152 3.000]	0.792	0.092 †
**Hopkins Verbal Learning Task delayed recall**	3.209 [0.569 6.049]	1.371	**0.041**
**Hopkins Verbal Learning Task total recall**	7.498 [3.207 11.679]	2.092	**0.007**
**Total FAS**	18.066 [10.484 25.944]	3.913	**0.001**
**Animal Fluency**	8.201 [5.076 11.378]	1.616	**0.001**
**Digit Symbol**	14.000 [6.807 21.094]	3.701	**0.001**
Rey Figure Copy	1.791 [−0.534 4.224]	1.204	0.161
**Rey Figure Immediate Recall**	8.749 [2.959 14.251]	2.940	**0.016**
**Rey Figure Delayed Recall**	6.764 [0.919 12.429]	2.923	**0.042**
**Graded Naming Test**	4.070 [1.280 6.999]	1.427	**0.012**
Normal Appearing White Matter volume (% in ICV)	0.291 [−0.637 1.274]	0.478	0.549
Cerebrospinal Fluid volume (% in ICV)	−0.984 [−2.395 0.413]	0.708	0.173
Left Hippocampal volume (% in ICV)	0.035 [0.002 0.067]	0.017	0.055
**Right Hippocampal volume (% in ICV)**	0.044 [0.016 0.072]	0.014	**0.005**
**Total Hippocampal volume (% in ICV)**	0.079 [0.026 0.132]	0.028	**0.009**
Volume of GM in Left Caudate (% in ICV)	0.009 [−0.022 0.040]	0.016	0.548
Volume of GM in Right Caudate (% in ICV)	0.028 [−0.0005 0.054]	0.014	0.062
Volume of GM in Left Putamen (% in ICV)	0.012 [−0.024 0.045]	0.017	0.494
Volume of GM in Right Putamen (% in ICV)	0.015 [−0.020 0.052]	0.018	0.430
Volume of GM in Left Globus Pallidus (% in ICV)	0.003 [−0.003 0.008]	0.003	0.382
Volume of GM in Right Globus Pallidus (% in ICV)	0.005 [−0.002 0.012]	0.003	0.129
Volume of GM in Left Thalamus (% in ICV)	−0.005 [−0.039 0.028]	0.017	0.801
Volume of GM in Right Thalamus (% in ICV)	−0.0008 [−0.034 0.031]	0.017	0.959
**Volume of the Parahippocampal Gyrus (% in ICV)**	0.076 [0.020 0.143]	0.031	**0.035**

*Note:* † p-value without bootstrap.

Legend: VSTM: Visual Short Term Memory, GM: Grey Matter, ICV: Intracranial Volume.

#### 3.2.2 Group comparison between imaging variables

**Fig. 2:**
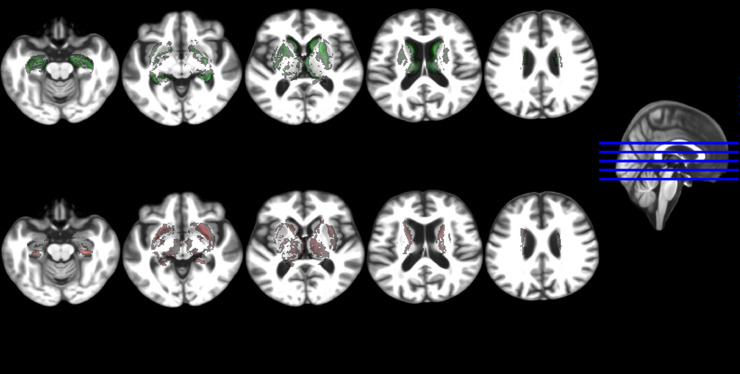
Five axial slices showing the voxel-wise differences between the probability distribution maps of the regions of interest analysed in the control group vs. the MCI group, all mapped in an age-relevant common template. The upper row shows in green the voxels where these differences (i.e. control map minus MCI map) are positives and the bottom row shows in red the voxels where these are negative.

Group comparison of imaging markers in MCI and control groups yielded significant differences in right hippocampal, total hippocampal, and parahippocampal gyrus volumes and no significant differences in other subcortical areas (i.e. all corrected by head size, Table 2, Supplementary Figure S2). Fig. 2 illustrates the results of subtracting the probability maps of the distribution of the ROIs analysed in the MCI group from those in the control group (i.e. voxel-wise group differences) in five representative axial slices. Despite global atrophy measurements (i.e. CSF and NAWM volumes) not differing between both groups, the control group had higher probability distribution of the GM in these structures around the ventricles of the template compared to the probability distribution of the GM in these structures in the MCI group (Fig. 2). Despite this being indicative of wider ventricles in the MCI group with respect to the control group, a closer look at the ventricular deformation patterns suggest these differences being due to individual morphometry differences possibly related to lesion load and abnormalities (not analysed).

### 3.3 Bivariate relations

#### 3.3.1 Bivariate relations in each cognitive group

The pattern of bivariate bootstrapped correlations differed in control and MCI groups (Tables 3 and 4). Amongst cognitive variables, however, the outcome from the Rey figure immediate and delayed recalls, significantly correlated in both groups even after FDR correction (Table 3). Statistically significant correlations at the 0.01 level (2-tailed) were consistent regardless of whether missing values were excluded list-wise or pair-wise.

**Table 3:** Bivariate bootstrapped Pearson's correlations (r) between the results of the cognitive tests, separately, in control (upper triangle) and MCI (bottom triangle) groups.Significance levels: * *P*<0.05, ** *P*<0.001. Results underlined survived FDR correction. Missing values were excluded pair-wise.

	(A)	(B)	(C)	(D)	(E)	(F)	(G)	(H)	(I)	(J)	(K)	(L)	(M)	(N)	(O)
(A)	1	0.499*	0.389	0.625^⁎⁎^	−0.828^⁎⁎^	0.213	0.386	0.287	0.313	0.413*	0.462*	0.140	0.479*	0.464*	0.651^⁎⁎^
(B)	0.183	1	0.572*	0.230	−0.365	0.292	0.262	0.247	0.300	0.315	0.139	0.067	0.274	0.421*	0.321
(C)	0.452	0.586*	1	0.083	−0.516*	0.183	0.257	0.168	−0.004	0.156	0.310	0.164	0.219	0.391	0.284
(D)	0.261	0.470*	0.530*	1	−0.357	0.244	0.310	0.301	0.295	0.490*	0.325	0.436*	0.602*	0.405	0.402
(E)	−0.003	−0.183	−0.041	−0.120	1	−0.175	−0.260	−0.274	−0.299	−0.207	−0.589*	−0.120	−0.353	−0.326	−0.615^⁎⁎^
(F)	−0.021	0.248	0.347	0.525*	−0.045	1	0.587*	0.668^⁎⁎^	0.040	0.265	0.188	0.075	0.164	0.403	0.142
(G)	0.431	0.271	0.371	0.767^⁎⁎^	−0.189	0.471*	1	0.469*	0.237	0.487*	0.299	−0.045	0.218	0.445*	0.315
(H)	0.326	0.574*	0.400	0.808^⁎⁎^	−0.334	0.306	0.731^⁎⁎^	1	0.085	0.413*	0.215	0.052	0.297	0.384	0.139
(I)	−0.340	0.091	0.444	0.257	0.055	0.064	0.009	0.248	1	0.345	0.277	−0.167	0.139	0.102	0.301
(J)	−0.206	0.209	0.151	0.342	−0.428	−0.134	0.316	0.529*	−0.052	1	0.320	0.187	0.488*	0.373	0.275
(K)	0.203	0.416	0.192	0.638*	−0.477*	0.380	0.393	0.485*	0.060	0.222	1	−0.096	0.346	0.396	0.664^⁎⁎^
(L)	0.075	0.070	0.292	0.179	−0.060	0.466*	0.105	0.019	0.036	−0.291	0.109	1	0.585*	0.382	−0.030
(M)	0.651*	0.096	0.426	0.567*	0.147	0.513*	0.670^⁎⁎^	0.416	0.017	−0.201	0.102	0.379	1	0.785^⁎⁎^	0.562*
(N)	0.594*	0.152	0.477	0.624*	0.114	0.558*	0.647*	0.397	0.100	−0.092	0.209	0.384	0.911^⁎⁎^	1	0.707^⁎⁎^
(O)	0.014	0.406	0.195	0.484*	0.074	−0.002	0.178	0.407	0.099	0.178	0.358	0.008	0.061	0.014	1

Legend: (A) Short term memory shape-only with 3 objects, (B) Short term memory shape-colour binding with 3 objects, (C) Short term memory shape-colour binding with 2 objects, (D) Addenbrooke's Cognitive Examination, (E) Trail Making Task B-A, (F) Hopkins Verbal Learning Task recognition, (G) Hopkins Verbal Learning Task delayed recall, (H) Hopkins Verbal Learning Task total recall, (I) Total FAS, (J) Animal fluency, (K) Digit symbol, (L) Rey figure copy, (M) Rey figure immediate recall, (N) Rey figure delayed recall, (O) Graded naming test.

**Table 4:** Bivariate bootstrapped Pearson's correlations (r) between the imaging parameters, separately, in control (upper triangle) and MCI (bottom triangle) groups.Significance levels: * *P*<0.05, ** *P*<0.001. Results underlined survived FDR correction. Missing values were excluded pair-wise.

	(1)	(2)	(3)	(4)	(5)	(6)	(7)	(8)	(9)	(10)	(11)	(12)	(13)	(14)
(1)	1	−0.745^⁎⁎^	0.273	0.374	0.352	0.293	0.417*	0.337	0.099	0.460*	−0.190	0.495*	0.486*	0.131
(2)	−0.527*	1	−0.430*	−0.677^⁎⁎^	−0.599*	−0.486*	−0.634^⁎⁎^	−0.471*	−0.156	−0.569*	0.091	−0.500*	−0.510*	−0.295
(3)	−0.064	−0.310	1	0.654^⁎⁎^	0.922^⁎⁎^	0.396	0.389	0.180	0.320	0.331	0.303	0.398*	0.449*	0.227
(4)	0.051	−0.071	0.806^⁎⁎^	1	0.900^⁎⁎^	0.457*	0.354	0.221	0.130	0.284	0.012	0.400*	0.393	0.422*
(5)	−0.008	−0.203	0.952^⁎⁎^	0.949^⁎⁎^	1	0.466*	0.409*	0.219	0.254	0.340	0.184	0.439*	0.465*	0.349
(6)	−0.256	−0.130	0.570*	0.521*	0.574*	1	0.764^⁎⁎^	0.698^⁎⁎^	0.343	0.281	−0.0003	0.685^⁎⁎^	0.673^⁎⁎^	0.336
(7)	−0.243	0.140	0.578*	0.623*	0.631*	0.831^⁎⁎^	1	0.697^⁎⁎^	0.349	0.533*	0.0322	0.554*	0.671^⁎⁎^	0.313
(8)	−0.258	−0.200	0.0514	0.003	0.029	0.336	0.193	1	0.740^⁎⁎^	0.208	−0.010	0.655^⁎⁎^	0.667*	−0.022
(9)	−0.067	−0.120	0.233	0.269	0.264	0.302	0.399	0.696^⁎⁎^	1	0.083	0.254	0.547*	0.605*	−0.175
(10)	−0.503*	0.0009	0.182	0.088	0.143	0.306	0.272	0.444*	0.425	1	0.401	0.224	0.435*	0.382
(11)	−0.365	0.107	0.288	0.152	0.233	0.338	0.433	0.391	0.554	0.585*	1	−0.086	0.164	−0.006
(12)	−0.430	0.050	0.594*	0.645*	0.651*	0.573*	0.502*	0.112	0.051	0.280	0.310	1	0.858^⁎⁎^	0.285
(13)	−0.400	−0.082	0.702^⁎⁎^	0.635*	0.704^⁎⁎^	0.639*	0.609*	0.128	0.091	0.407	0.396	0.934^⁎⁎^	1	0.313
(14)	−0.010	−0.342	0.669*	0.588*	0.659*	0.360	0.422	0.236	0.429	0.309	0.539*	0.615*	0.619*	1

Legend: (1)% of Normal-appearing white matter volume in ICV, (2)% of CSF volume in ICV, (3)% Left hippocampal volume in ICV, (4)% Right hippocampal volume in ICV, (5)% Total hippocampal volume in ICV, (6)% Left caudate nucleus grey matter volume in ICV, (7)% Right caudate nucleus grey matter volume in ICV, (8)% Left putamen grey matter volume in ICV, (9)% Right putamen grey matter volume in ICV, (10)% Left globus pallidus grey matter volume in ICV, (11)% Right globus pallidus grey matter volume in ICV, (12)% Left thalamus grey matter volume in ICV, (13)% Right thalamus grey matter volume in ICV, (14)% parahippocampal volume in ICV.

##### 3.3.1.1 Bivariate relations between cognitive variables

After FDR correction, the outcome from the STM binding task that presented 3 Shape-only objects correlated with the outcome from the trail making test and graded naming test in the control group. The STM binding tasks that presented 2 and 3 Shape-Colour objects only correlated with each-other in the control group. None of these tasks correlated with any other cognitive variable in the MCI group after FDR correction.

##### 3.3.1.2 Bivariate relations between imaging variables

In the control group general atrophy and GM in thalami correlated with all other imaging volumes except those from the globus pallidus and the parahippocampi. In fact, in this group the GM volume in the right globus pallidus did not correlate with any other imaging volume and the parahippocampal volume only correlated with the right hippocampal volume. On the contrary, in the MCI group general atrophy measurements did not correlate with any other structural volumes and the parahippocampal volume correlated with all hippocampal, right globus pallidus and thalamic measurements. The thalami GM volume in the MCI group also correlated with the hippocampal and caudate volumes (Table 4).

##### 3.3.1.3 Bivariate relations between cognitive and imaging variables

Global atrophy measurements only marginally correlated with the outcome from the digit symbol test in controls (NAWM: *r* = 0.42,*P* = 0.035; CSF: *r*=−0.45,*P* = 0.024) but this correlation disappeared after FDR correction. In MCI patients, however, NAWM volume correlated with Rey figures immediate recall (*r* = 0.58,*P* = 0.006). In this group, its correlation with HLVT recognition (*r* = 0.55,*P* = 0.012) and HLVT delayed recall (*r* = 0.53,*P* = 0.012) did not survive multiple hypothesis testing. Also in the MCI group, CSF volume negatively correlated with HLVT delayed recall (*r* = −0.63,*P* = 0.002) and ACE (*r* = 0.60,*P* = 0.004). Its apparent correlation with the Rey figures immediate recall (*r* = −0.50,*P* = 0.022) did not survive FDR correction.

The hippocampal volume did not correlate with any cognitive variable in the control group, but correlated with the Shape Only Condition of STM task in the MCI group. After FDR correction only the left hippocampal volume remained correlated with this test in the MCI group. The STM binding condition with memory load 2 did not correlate with any imaging variable in the control group, but correlated with the GM volume in the left globus pallidus and with the parahippocampal volume in the MCI group (Supplementary Table S3). The former (i.e. STM binding with two objects and left globus pallidus volume in the MCI group) survived FDR correction (Supplementary data spreadsheet “Correlations_FDR_results.xlsx”). The STM binding condition with memory load 3 did not correlate with any imaging variable in either group.

##### 3.3.1.4 Bivariate relations of cognitive and imaging variables with demographic variables

In the control group, age only correlated with animal fluency (*r* = −0.41, *P* = 0.040), whereas in the MCI group age correlated with STM binding test with memory load 3 (*r* = −0.49, *P* = 0.020) and digit symbol (*r* = −0.46, *P* = 0.027). Age did not correlate with any of the imaging variables in the control group, but in the MCI group, age negatively correlated with the GM volumes in the hippocampus, caudate and thalami with similar strength (*r* ≈ −0.50) and significance (*P* < 0.050) levels. Only the correlation of the right thalamus with age survived FDR correction.

#### 3.3.2 Bivariate relations in the whole sample

##### 3.3.2.5 Bivariate relations between cognitive and imaging variables

The outcome from both conjunctive STM binding tasks (i.e. shape-colour with 2 and 3 objects) correlated with the volumes of total normal appearing white matter (*r* = 0.32, *P* = 0.047), and GM in the right caudate nucleus (*r* = 0.33, *P* = 0.030), but these correlations disappeared after bootstrap. The Shape Only condition significantly correlated (*r* > 0.42, *P* < 0.001) with all hippocampal volume measurements (i.e., left, right and total) before and after bootstrap. The HLVT total recall, digit symbol and Rey figure delayed recall tests were the cognitive tests that exhibited the widest pattern of correlations with imaging variables, with the digit symbol and Rey figure delayed recall showing correlation with total brain atrophy (i.e.% of CSF in ICV, *r* = 0.49, *P* = 0.001 and *r* = 0.41, *P* = 0.006 respectively) (Supplementary Table S4).

##### 3.3.2.6 Bivariate relations of cognitive and imaging variables with demographic variables

Age significantly correlated with structural volume deficits in left hippocampus (*r* = −0.35, *P* = 0.018), total hippocampus (borderline) (*r* = −0.29, *P* = 0.050), left thalamus (*r*=−0.32, *P* = 0.030) and right thalamus (*r*=−0.41, *P* = 0.005), which disappeared after bootstrap, and showed no correlation with any cognitive assessment scores. For years of education, significance was found with GM volumes of the right globus pallidus (*r* = −0.30,*P* = 0.043) and right thalamus (*r* = −0.33, *P* = 0.023), which also disappeared after bootstrap, and with the cognitive scores of HVLT recognition (*r* = 0.34, *P* = 0.025), HVLT delayed recall (*r* = 0.32, *P* = 0.029), digit symbol (*r* = 0.32, *P* = 0.030), Rey figure copy (*r* = 0.40, *P* = 0.014), graded naming (*r* = 0.34, *P* = 0.21), ACE (*r* = 0.48, *P* = 0.001), TMT B-A (*r*=−0.38, *P* = 0.009), total FAS (*r* = 0.45, *P* = 0.002), Rey figure immediate recall (*r* = 0.42, *P* = 0.004), and Rey figure delayed recall (*r* = 0.43, *P* = 0.004). After bootstrap, years of education and the cognitive variables mentioned remained correlated in addition to animal fluency (*r* = 0.457, *P* = 0.007, SE = 0.135).

### 3.4 Associations between cognitive and imaging variables

In the control group, regression analyses found the following associations: GM in the left caudate nucleus with the outcome from the graded naming test (*B* = 40.18, SE = 17.58, β = 0.43, *P* = 0.034), GM in the left globus pallidus with that from animal fluency (*B* = −235.44, SE = 107.26, β = −0.43, *P* = 0.04), GM in both thalami with results from the graded naming test (*B* = 36.85, SE = 16.90, β = 0.40, *P* = 0.041 and *B* = 46.83, SE = 18.07, β = 0.42, *P* = 0.017 respectively) and digit symbol (*B* = 189.56, SE = 58.93, β = 0.62, *P* = 0.004 and *B* = 212.38, SE = 65.19, β = 0.64, *P* = 0.004 respectively). The STM binding tasks were not associated with any imaging variable in this group.

**Fig. 3:**
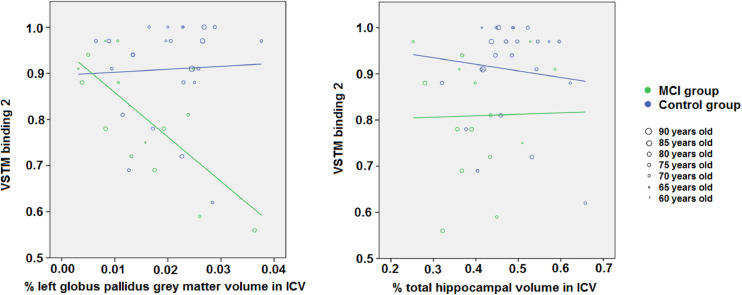
Associations of the VSTM binding test 2 with grey matter volume in the left globus pallidus and hippocampus, per group and accounting for age.

In the MCI group, global brain atrophy indicators were associated with the outcomes from the HVLT delayed recall and Rey figure immediate and delayed recall tests. Associations between total normal appearing white matter and the outcome of the HVLT delayed recall was (*B* = 1.62, SE = 0.62, β = 0.55, *P* = 0.019), and with the Rey figure immediate and delayed recall (*B* = 5.28, SE = 1.58, β = 0.73, *P* = 0.004) and (*B* = 4.53, SE = 1.58, β = 0.65, *P* = 0.012) respectively. Cerebrospinal fluid's association with the HVLT delayed recall was (*B* = −1.15, SE = 0.46, β = −0.53, *P* = 0.025), and with the Rey figure immediate and delayed recall (*B* = −3.02, SE = 1.32, β = −0.56, *P* = 0.036) and (*B* = −2.71, SE = 1.26, β = −0.53, *P* = 0.048) respectively. Its association with the visual STM binding task with 3 items had only borderline significance (*B* = 0.03, SE = 0.01, β = 0.44, *P* = 0.050). The associations found between the GM volumes in the subcortical structures and the results of the cognitive tests in this group (i.e. MCI) are shown in Table 5. The STM binding test with memory load of 2 was only associated with the GM volume in the left globus pallidus (β = −0.56, *P* = 0.042), but this association became non-significant (*P* = 0.057) after applying bootstrap considering a sample size of *n* = 1000. Of note, if the regression model only has age and gender as covariates (i.e. not adjusting for years of education), this association remains significant even after bootstrap (*B* = −9.034, SE = 0.17, 95%CI = [−14.43 −1.94], *P* = 0.012). Fig. 3 illustrates the regression slope for all study participants (i.e. regardless of biological sex differences) from both cognitive groups accounting for age. No significant association was found between the STM binding condition with memory load 2 and hippocampal volume (Fig. 3).

**Table 5:** Associations of the subcortical grey matter volumes adjusted by head size, with the cognitive tests in the MCI group, at P-values below 0.050, corrected for age, gender and years of education. Bootstrap results are based on 1000 bootstrap samples.

Subcortical structure	Cognitive test	Unstandardized coefficients		Std. coefficient	P-value	Associated in Control group (Yes/No)	Associated after applying bootstrap (Yes/No) (95% confidence interval; P)
		B	Std. error (SE)	β			
Left Hippocampus	STM shape-only (3 items)	1.007	0.385	0.688	0.021	No	Yes (95%CI = [0.250 1.706]; *P* = 0.017)
	Animal Fluency	−59.165	26.980	−0.560	0.043	No	No (95%CI = [−132.576 −2.858]; *P* = 0.084)
	Rey figure delayed recall	104.813	46.959	0.589	0.041	No	No (95%CI = [−16.763 183.874]; *P* = 0.053)
Right Hippocampus	Rey figure delayed recall	100.559	41.335	0.559	0.028	No	Yes (95%CI = [3.219 198.001]; *P* = 0.039)
Left Caudate	Rey figure delayed recall	127.168	51.214	0.697	0.025	No	Yes (95%CI = [2.573 213.555]; *P* = 0.015)
Left Putamen	ACE	71.997	32.189	0.448	0.040	No	Yes (95%CI = [16.304 136.525]; *P* = 0.015)
	HVLT total recall	51.774	22.851	0.496	0.038	No	No (95%CI = [1.722 121.651]; *P* = 0.093)
	Graded naming test	49.285	20.058	0.612	0.026	No	Yes (95%CI = [4.270 81.389]; *P* = 0.021)
	Graded naming test	47.468	17.466	0.582	0.015	No	Yes (95%CI = [8.266 79.555]; *P* = 0.020)
Left Globus Pallidus	STM shape-colour binding (2 items)	−7.671	3.301	−0.563	0.042	No	No (95%CI = [−12.678 9.190]; *P* = 0.057)
Right Putamen	ACE	−76.274	34.474	−0.502	0.042	No	No (95%CI = [−146.223 8.104]; *P* = 0.056)

## 4 Discussion

This study was set out to investigate the extent to which impaired abilities to process conjunctive feature bindings in STM in patients with cognitive decline seemingly due to neuroprogressive diseases could be accounted for by structural changes in regions of the medial temporal lobe (i.e., the hippocampus and parahippocampal gyrus) or striatal regions which remain unexplored in the context of this new memory paradigm. Our key findings were (1) no apparent association between hippocampal GM volume and impaired abilities to process bound information in STM (2) GM volume of regions of the striatum (i.e., globus pallidus) can account for such impairments specifically, when memory load allows for a good discrimination between patients and controls and premorbid cognition is not considered. We discuss these findings in turn.

The fact that the pattern of bivariate correlations, mainly between imaging variables, differed in control and MCI groups is an interesting result, especially the fact that general brain atrophy correlated with atrophy in hippocampi and caudate in the control group but not in the MCI group. This, however, is in-line with findings from other studies that have found spatially complex atrophy patterns in MCI, consistently involving cortical and periventricular regions and not subcortical grey matter (e.g., Fan et al., 2008). A systematic review and meta-analysis on cerebral atrophy in MCI concluded that although the medial temporal lobe is likely to be more vulnerable to MCI, atrophy in this brain region represents a relatively small proportion of the whole brain loss, suggesting that the source of unaccounted volume loss in MCI requires further investigation (Tabatabaei-Jafari et al., 2015). Being age known to correlate with general atrophy in cognitively normal ageing individuals (Royle et al., 2013), then, not surprisingly the bivariate correlations between age and the volumetric imaging parameters analysed differed between groups despite both being age-matched.

In agreement with other studies, the GM volume in the hippocampus and in the parahippocampal gyrus, significantly differed between controls and MCI patients. However, in both groups the hippocampal and parahippocampal volumes were correlated. The GM volume in basal ganglia structures, however, failed to distinguish between groups. Atrophy of basal ganglia structures has been linked with cognitive deficits in different types of dementia (e.g., AD, Parkinson's and Huntington's disease) but, to a lesser extent, with MCI (Cho et al., 2014). As of yet, the relatively sparse research observing globus pallidus volumes in individuals with AD appears to have found no significant differences compared with volumes of healthy controls, despite pallidal volume reductions being observed in fronto-temporal dementia patients (Moller et al., 2015; Yi et al., 2016). The possibility that the MCI individuals in this sample would be displaying early signs of fronto-temporal dementia rather than AD seems unlikely, especially as the binding task has shown high specificity to AD (Della Sala, Parra, Fabi, Luzzi, and Abrahams, 2012; Cecchini et al., 2017). Hence, the fact that the group of MCI patients presented with hippocampal atrophy would represent an increasing likelihood of risk of AD pathology despite hippocampal atrophy *per se* lacks specificity for AD, as it can be also present in non-AD forms of dementia (Pini et al., 2016). Hippocampal atrophy in relation to AD and cognitive decline with ageing is well documented and is thought to be one of the earliest volumetric biomarkers of the disease, in line with volume differences observed here (Arlt et al., 2013) and findings in rodent models of the disease (Redwine et al., 2003). The role of the parahippocampal gyrus in cognitive processes is also well known (Aminoff et al., 2013) and its atrophy has been recently noted in the context of age-related cognitive decline (Karama et al., 2014), although the same study warns on the lifelong effect of childhood cognition in the preservation (or thinning) of the cortex.

In the context of the present study, we failed to find associations between hippocampal volume and impaired conjunctive STM binding functions in patients with MCI. There was an association between hippocampal volume and shape-only processing in MCI patients. The apparent involvement of the hippocampus in working memory tasks when memory load increases (Parra et al., 2019) has been recently noted. However, such involvement is not binding specific as neither STM binding conditions (2 or 3 items) revealed hippocampal dependant impairment. Evidence has now accumulated confirming that conjunctive STM binding, a function sensitive to AD, is independent from the hippocampus (Jonin et al., 2019; M.A. Parra et al., 2014; M.A. Parra et al., 2015). Here, using different techniques, we demonstrate that this is also true in older people with cognitive impairment who are at a high risk of developing AD dementia. (Parra et al., 2015) noted, that whereas conjunctive STM binding deficits in familial AD were accounted for by loss of white matter integrity in the prefrontal cortex, damage to white matter in medial temporal lobe (i.e., fornix) accounted for poor performance on LTM relational task (i.e., PAL of Wechsler Memory Scale). Additionally, contrary to conjunctive STM binding, which seems to rely on a more localized network, associative memory seems to involve an extended network. In the present study we found that global brain atrophy indicators were associated with the outcomes from the HVLT delayed recall. The HVLT assesses the ability to form categorical clusters which is a form of relational memory in the semantic domain (Calia et al., 2017). Such a reliance on wide brain network renders relational memory tests more vulnerable to a range of neuropsychiatric diseases and as a result less specific. STM binding has proved to be a specific AD marker (Cecchini et al., 2017; Della Sala et al., 2012; M.A. Parra et al., 2010), a property seemingly resulting from the more focal network subserving this function.

Our regression analysis results indicate that the GM volume of the globus pallidus can be associated with STM binding performance in MCI patients. This result adds strength to the argument that the search for a conjunctive STM binding biomarker should shift focus away from the hippocampus (Hoefeijzers, Calia, & Parra et al., 2016; Jonin et al., 2019), and possibly towards structures within the basal ganglia. Initially, only two imaging variables showed significant bivariate correlations with the STM binding tasks in our whole sample: the GM volume in the right caudate nucleus and the total normal appearing white matter. Volume changes with cognitive decline in both have been previously described with respect to AD (Persson et al., 2018). However, involvement of basal ganglia structures such as the caudate nucleus (associated with working memory functions), or normal appearing white matter (commonly associated with processing speed functions) in conjunctive STM binding is a new finding. After adjusting for demographic variables (age and biological sex) and years of education, the bivariate correlations initially found with the results from the visual STM binding tasks disappeared, and in the MCI group, the visual STM binding task with 2 objects was associated with the GM volume in the globus pallidus. In addition, the same cognitive task with 3 objects was associated with the total brain tissue atrophy given by the percentage of CSF volume in ICV. But the latter association (i.e., STM binding task with 3 objects), if replicated in larger samples, needs to be interpreted with caution as patients’ performance on this task was close to chance (see Parra et al., 2019) for suggestions about optimal task settings). These results seem to be in line with recent findings by (Parra et al., 2019). The authors suggested that STM binding is extremely sensitive to the early stages of AD and such sensitivity leads to near floor performance as individuals approach the dementia stages. This could explain why in the condition when STM binding was assessed with 3 items a non-specific pattern of correlation involving extensive brain regions and structures was found. However, when the task demands do not excessively tax memory functions (see (Logie et al., 2015)), as it is the case with visual arrays of 2 coloured shapes, the impaired STM binding abilities, which were significant in MCI patients, were accounted for by more specific neuropathological findings. Currently, there has been little exploration of the role of the globus pallidus in STM, and to the best of our knowledge the role of basal ganglia structures in conjunctive STM binding has not been researched. The current view, based on pallidotomy studies, is that the globus pallidus has no role in STM function (Birska et al., 2016) in patients with dystonia.

Animal studies have found that the globus pallidus is involved in memory processing. For example, a study found that excitotoxic lesions in the globus pallidus, which spare the passing fibres, lead to impairments in acquiring and remembering light-reward conditioning but leaving basic visual discrimination intact (Evenden et al., 1989). The patients assessed here showed completely normal visual discrimination as assessed by a perceptual binding task used as a screening tool prior to the STM binding test. The impaired visual conditioning in rats has been linked with the cholinergic neuronal loss in the globus pallidus (Everitt et al., 1987). Other animal experiments have shown that ventral pallidum sends dense inhibitory projections to the mediodorsal thalamus (Churchill et al., 1996), an area that is required for and is active during working-memory tasks (Han et al., 2013; Stokes and Best, 1990). Pharmacologically activating ventral pallidum impairs STM in an alternating task in the T-maze (Kalivas et al., 2001), as well as in a delayed non-matching-to-lever task (Zhang et al., 2005). At the circuit-level, globus pallidus is linked to the prefrontal cortex via thalamus and together is critical for short-term memory processing (Dunnett, 1990). Although it is yet to develop a corresponding task in rodents for VSTMB, the recognition component of this task can be compared with object recognition tasks in rodents. In fact, recent studies relying on EEG based methods have confirmed that STM binding relies on a cortical network involving frontal, parietal, and occipito-temporal regions (Parra et al., 2017; Pietto et al., 2016; Smith et al., 2017). Globus pallidus lesions impair the short-term object recognition (Ennaceur, 1998). Combining the touch-screen technology (Talpos et al., 2014) with pharmacological or optogenetic inactivation (Goshen, 2014), future rodent studies will allow further substantiation of the causal role of globus pallidus in memory binding. Hence, this study adds to the existing knowledge about the neural correlates of STM binding and provides evidence suggesting that in cognitively impaired older adults with a high-risk profile of AD, loss of ability to conjunctively bind information in STM can be accounted for by damage to striatal regions such as the globus pallidus.

A potential account for why the more taxing condition (STM binding with 3 items) was less discriminative in the present study was already provided (Parra et al., 2019). However, it is worth noting that in the present study the average performance of the control group was also low in this condition of the STM task. Based on existing evidence about the very early impact of AD on this cognitive functions (Parra et al., 2010; Parra et al., 2011), it might be plausible to suggest that a few participants of the control group could be already in the preclinical stages of AD (see Koppara et al., 2015; Parra et al., 2017). Future studies are needed to elucidate whether and how poor STM binding performance should be used as a criterion for group membership. Regarding our key neuroimaging finding, it is likely that results of previous studies, which show no reduction in globus pallidus volumes, have focused on total structural volume as opposed to its grey matter content assessed here. This would explain the volume differences observed between groups here, and possibly explain why we see no significant reduction in other basal ganglia volumes.

Using striatal signal as a marker of advanced amyloidosis may increase predictive power in AD research (Hanseeuw et al., 2018). Assuming the globus pallidus is implicated in binding (or in cognitive functions underpinned by this cognitive construct), the inner architecture, circuitry and downstream centres involved in this task are still to be elucidated. Degeneration of nigrostriatal inputs to the globus pallidus involved in Parkinson's disease seems to be unlikely as Della Sala et al. (2012) found that conjunctive STM binding could distinguish AD from other forms of dementia including Parkinson's dementia. Perhaps instead, other major projections to striatal structures whose degeneration acts as hallmarks of AD pathology should be investigated (see Hanseeuw et al., 2018). For example, the ventral visual stream, which has an established role in binding function and heavily supplies the perirhinal cortex previously implicated in binding (Staresina and Davachi, 2010). Nevertheless, previous studies that have linked the perirhinal cortex with binding functions, have failed to link this region with conjunctive object colour binding (Clarke and Taylor, 2014), a result that requires to be explored in larger studies.

The strengths of our study are the comprehensive battery of cognitive tests and the state-of-art image processing followed by a detailed visual scrutiny and manual boundary rectification, resulting in accurate measurements. Different from the vast majority of studies, we did not analyse the volume of the subcortical structures, but the volume of grey matter in them, reducing the impact of inaccuracies that could arise in the boundary delineation of these structures. The protocol used in this study also described hippocampal measurements as inaccurate in 2/3 cases when not corrected manually (Wardlaw et al., 2011). A study by Meijerman et al. (2018), looked at the reproducibility of deep grey matter atrophy assessment in large data sets, including data on the globus pallidus, and found substantial errors in reproducibility. We accounted for the possible inaccuracies in the results caused by this specific sample that may not be fully representative of the population using bootstrap with *n* = 1000. The application of this technique yielded an important finding worth exploring further: if accounted for years of education, the apparent effect of the globus pallidus in the conjunctive STM binding might disappear. This result constitutes another evidence supporting the lifelong effect of prior cognition in the apparent associations that might be found between brain structural changes and cognition in late adulthood (Karama et al., 2014). In addition to the small sample size, the fact that brain abnormalities were not assessed is a caveat in our study. Research has shown that brain iron deposits and white matter hyperintensities are associated with lifetime reductions in cognitive ability and cognitive aging (Valdés Hernández et al., 2016). In fact, there is evidence that age related mineral deposition is particularly prevalent in the globus pallidus (Glatz et al., 2013). Here, age was controlled for in the analyses, but it would be important to detail associated regional lesions and mineral deposition in future studies to ensure that our findings are not confounded by these factors.

The results presented here open a new avenue in the biological substrate underpinning conjunctive STM binding- the globus pallidus. We have strengthened the argument that the hippocampus is not involved in conjunctive STM binding and have demonstrated a possible role for structures within the basal ganglia. We propose that search for the binding task's neural correlates shifts its focus away from medial temporal lobe structures and towards structures supporting context-free memory with the basal ganglia as promising candidate. Further study will first need to establish reproducibility, and then explore other possible structures involved with conjunctive STM binding. This could provide a better understanding of the mechanisms involved in AD progression, and lead to earlier and more precise disease diagnosis.

## Funding sources

This project is partially funded by the UK Biotechnology and Biological Sciences Research Council (BBSRC) through the International Partnership Award BB/P025315/1. MPR, CC, and SDS work was funded by the Alzheimer's Society (AS-SF-14-008). MCVH is funded by Row Fogo Charitable Trust (Grant No. BROD.FID3668413), MCVH received funds from the European Union Horizon 2020 [PHC-03–15, project No 666,881, ‘SVDs@Target’] and the Fondation Leducq Network for the Study of Perivascular Spaces in Small Vessel Disease [ref no. 16 CVD 05]. This work was also supported by the UK Dementia Research Institute which receives its funding from DRI Ltd, funded by the UK Medical Research Council, Alzheimer's Society and Alzheimer's Research UK.

## Authors contributions

Maria del C. Valdés Hernández: Analysis design, image processing (i.e. image data generation and processing), statistical analysis, writing and revising the manuscript

Rupert Clark: Image processing (i.e. image data generation and processing), statistical analysis, editing, revision and approval of the manuscript

Szu-Han Wang, John Starr: Editing, revision and approval of the manuscript

Federica Guazzo^,^ Clara Calia: Cognitive assessment, cognitive data generation and analysis, editing, revision and approval of the manuscript

Sergio Della Sala: Primary study design, editing, revision and approval of the manuscript

Mario Alfredo Parra: Primary study design, patient recruitment, cognitive assessment, data analysis design, editing, revision and approval of the manuscript

Vivek Pattan: Patient recruitment, clinical assessment, editing, revision and approval of the manuscript.

**Sponsor's Role:** The sponsors did not participate in the design, methods, recruitment, data collections, analysis or preparation of this manuscript

The Striatum, the Hippocampus, and Short-Term Memory Binding: Volumetric Analysis of the Subcortical Grey Matter's Role in Mild Cognitive Impairment

## Declaration of Competing Intrest

None

## Appendix Supplementary materials

Image, application 1

Image, application 2

Image, application 3

Image, application 4

Image, application 5
