# Supplementary material for: The striatum, the hippocampus, and short-term memory binding: Volumetric analysis of the subcortical grey matter's role in mild cognitive impairment
Source: Neuroimage Clin. 2019 Dec 29;25:102158. doi: 10.1016/j.nicl.2019.102158 (PMC7036699; doi:10.1016/j.nicl.2019.102158)
Supplement: Supplementary file 1 [file mmc1.docx]

**The Striatum, the Hippocampus, and Short-Term Memory Binding: Volumetric Analysis of the Subcortical Grey Matter’s Role in Mild Cognitive Impairment**

**SUPPLEMENTARY MATERIAL**

Table S1. Structural MRI sequence details

| Sequence parameters | T1-weighted | T2-weighted | FLAIR | T2*weighted |
| --- | --- | --- | --- | --- |
| TR/TE/TI (ms) | 9.7/3.984/500 | 11320/102/- | 9000/140/2200 | 940/15/- |
| Pixel bandwidth (KHz) | 15.63 | 20.83 | 15.63 | 12.50 |
| Matrix | 192 x 192 | 256 x 256 | 256 x 192 | 256 x 192 |
| No. slices | 160 | 80 | 40 | 80 |
| Slice thickness (mm) | 1.3 | 2.0 | 4.0 | 2.0 |
| Voxel size (mm3) | 1 x 1 x 1.3 | 1 x 1 x 2 | 1 x 1 x 4 | 1 x 1 x 2 |

Table S2. Descriptive statistics of the variables involved in the analyses, split by gender

|  | | **Controls (mean+/-SD)** | | **Mild Cognitive Impairment (mean +/-SD)** | |  |
| --- | --- | --- | --- | --- | --- | --- |
|  |  | Female (n= 17) | Male (n= 8) | Female (n= 8) | Male (n= 13) | Total (n= 46) +/- SD |
| **Demographic Variables (years)** | Age | 75.9+/-4.4 | 78.0+/-7.2 | 70.2+/-3.9 | 76.3+/-5.3 | 75.2 +/- 5.5 |
|  | Education | 14.6+/-3.6 | 15.8+/-3.9 | 11.4+/-2.2 | 13.4+/-4.4 | 14.4 +/- 3.8 |
| **Imaging Variables (% in ICV)** | NAWM | 33.01+/-1.59 | 31.80+/-1.31 | 31.61+/-1.15 | 32.85+/-1.19 | 32.69 +/- 1.38 |
|  | CSF | 30.37+/-2.93 | 32.03+/-1.55 | 31.20+/-0.87 | 32.52+/-1.78 | 31.08 +/- 2.17 |
|  | Left Hippocampus | 0.2476+/-0.0456 | 0.2135+/-0.0376 | 0.2499+/-0.0512 | 0.1755+/-0.0170 | 0.2276 +/- 0.0491 |
|  | Right Hippocampus | 0.2459+/-0.0497 | 0.2376+/-0.0240 | 0.2257+/-0.0236 | 0.1856+/-0.0407 | 0.2285 +/-0.0473 |
|  | Total Hippocampus | 0.4935+/-0.0888 | 0.4511+/-0.0521 | 0.4755+/-0.0737 | 0.3611+/-0.0548 | 0.4561 +/- 0.0903 |
|  | Left Caudate Nucleus | 0.2044+/-0.0317 | 0.1586+/-0.0572 | 0.2225+/-0.0411 | 0.1565+/-0.0382 | 0.1865 +/- 0.0450 |
|  | Right Caudate Nucleus | 0.2141+/-0.0343 | 0.1927+/-0.0312 | 0.2204+/-0.0305 | 0.1544+/-0.0381 | 0.1950 +/- 0.0443 |
|  | Left Putamen | 0.2466+/-0.0514 | 0.2032+/-0.0459 | 0.2510+/-0.0500 | 0.1960+/-0.0459 | 0.2259 +/- 0.0502 |
|  | Right Putamen | 0.2449+/-0.0551 | 0.2351+/-0.0385 | 0.2588+/-0.0361 | 0.1951+/-0.0557 | 0.2331 +/- 0.0493 |
|  | Left Globus Pallidus | 0.01980+/-0.00671 | 0.01770+/-0.00750 | 0.02020+/-0.00670 | 0.01100+/-0.00610 | 0.01726 +/-0.00836 |
|  | Right Globus Pallidus | 0.02050+/-0.01128 | 0.01623+/-0.00843 | 0.02150+/-0.00770 | 0.00858+/-0.00621 | 0.01577 +/- 0.00981 |
|  | Left Thalamus | 0.2528+/-0.0433 | 0.2094+/-0.0426 | 0.2839+/-0.0478 | 0.2271+/-0.0251 | 0.2412 +/- 0.0457 |
|  | Right Thalamus | 0.2441+/-0.0357 | 0.2065+/-0.0474 | 0.2814+/-0.0478 | 0.2054+/-0.0156 | 0.2331 +/- 0.0459 |
|  | Parahippocampal Gyrus | 0.2898+/-0.0959 | 0.2844+/-0.1775 | 0.2561+/-0.0541 | 0.1874+/-0.0451 | 0.2516 +/- 0.0949 |
| **Cognitive Assessment Scores** | STM Shape-only 3 | 0.8787+/-0.0844 | 0.8817+/-0.0557 | 0.8620+/-0.0773 | 0.8071+/-0.0553 | 0.8665 +/- 0.0759 |
|  | STM Binding 3 | 0.7167+/-0.0863 | 0.6400+/-0.0609 | 0.6740+/-0.1011 | 0.6214+/-0.1258 | 0.6777 +/- 0.1035 |
|  | STM Binding 2 | 0.9033+/-0.1130 | 0.8857+/-0.1143 | 0.7880+/-0.1270 | 0.8271+/-0.1116 | 0.8705 +/- 0.0254 |
|  | ACE (*dementia screening, orientation, attention, memory, language, visuospatial abilities*) | 94.87+/-4.72 | 94.50+/-6.47 | 86.80+/-9.01 | 81.86+/-8.91 | 89.24 +/- 8.55 |
|  | TMT B-A (*speed of processing and executive functioning*) | 58.33+/-51.88 | 65.67+/-26.17 | 71.40+/-48.09 | 82.43+/-21.47 | 66.65 +/- 40.45 |
|  | HVLT Recognition (*dementia screening, memory*) | 11.13+/-1.41 | 8.50+/-2.88 | 7.60+/-2.30 | 9.86+/-1.95 | 9.55 +/- 2.34 |
|  | HVLT Delayed Recall (*dementia screening, memory*) | 7.87+/-3.64 | 5.83+/-4.67 | 4.60+/-4.88 | 3.86+/-3.58 | 5.76 +/- 4.00 |
|  | HVLT Total Recall (*dementia screening, memory*) | 27.07+/-4.74 | 20.50+/-8.36 | 19.80+/-4.97 | 16.57+/-6.83 | 21.26 +/- 6.87 |
|  | Total FAS (*phonemic fluency*) | 48.80+/-15.09 | 43.00+/-7.21 | 27.00+/-5.00 | 30.00+/-12.52 | 40.65 +/- 15.29 |
|  | Animal Fluency (*semantic fluency*) | 20.33+/-4.72 | 19.33+/-3.72 | 13.20+/-5.40 | 11.00+/-4.97 | 15.70 +/- 6.48 |
|  | Digit Symbol (*speed of processing and executive functioning*) | 57.40+/-15.94 | 52.50+/-7.94 | 46.80+/-5.76 | 37.95+/-8.95 | 49.98 +/- 13.44 |
|  | Rey Figure Copy (*visuospatial abilities, executive functioning, visual memory*) | 33.43+/-4.04 | 32.67+/-1.94 | 31.00+/-4.42 | 31.93+/-2.21 | 31.60 +/- 5.95 |
|  | Rey Figure Immediate Recall (*visuospatial abilities, executive functioning, visual memory*) | 19.60+/-7.31 | 17.83+/-7.41 | 11.70+/-9.91 | 10.14+/-9.45 | 15.16 +/- 8.98 |
|  | Rey Figure Delayed Recall (*visuospatial abilities, executive functioning, visual memory*) | 18.07+/-6.50 | 18.08+/-7.93 | 11.70+/-10.76 | 11.08+/-9.25 | 15.48 +/- 8.13 |
|  | Graded Naming Test (*naming*) | 22.53+/-4.67 | 23.33+/-2.88 | 19.60+/-4.62 | 17.71+/-4.27 | 21.28 +/-4.41 |

Legend: NAWM: normal appearing white matter, CSF: cerebrospinal fluid, VSTM: Visual Short Term Memory, ACE: Addenbrooke’s Cognitive Examination, TMT B-A: Trail Making Task B-A, HVLT: Hopkins Verbal Learning Task

Figure S1. Mean cognitive assessment scores in MCI and Control groups. N=46, significance in group differences is indicated as follows: *P<0.05, **P<0.01, ***P<0.001


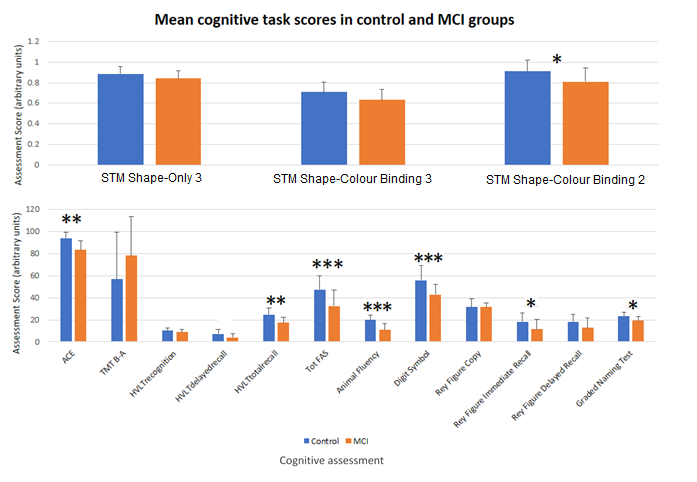


Figure S2. Mean volume of imaging markers in MCI and Control groups. N=46, significance in group differences is indicated as follows: *P<0.05, **P<0.01


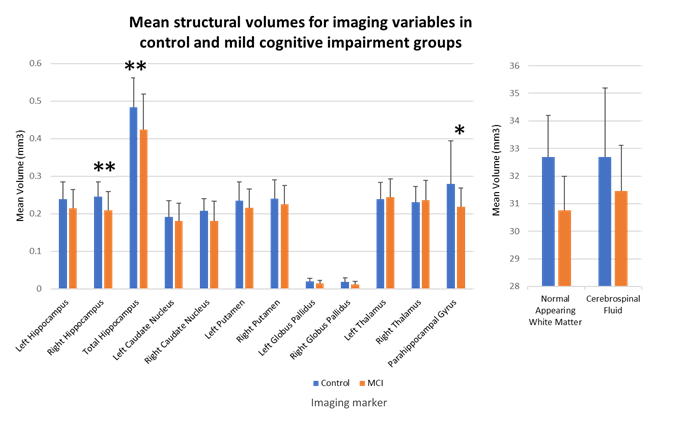


*Table S3. Bivariate bootstrapped Pearson's (r) correlations between imaging and cognitive variables (i.e. scores from the cognitive tests) per group (pairwise exclusion)*

|  | STM shape-only 3 | STM bind 3 | STM bind 2 | ACE | TMT B-A | HVLT recog-nition | HVLT delayed recall | HVLT total recall | Total FAS | Animal fluency | Digit Symbol | Rey Figure Copy | Rey Fig immed recall | Rey Fig delayed recall | Graded naming test |
| --- | --- | --- | --- | --- | --- | --- | --- | --- | --- | --- | --- | --- | --- | --- | --- |
| Control group | | | | | | | | | | | | | | | |
| (1) | .109 | .148 | .327 | -.108 | -.355 | .066 | .213 | .112 | .109 | -.036 | **.424*** | -.056 | .143 | .178 | .163 |
| (2) | -.133 | -.132 | -.136 | -.044 | .223 | .096 | -.135 | -.138 | -.009 | .122 | **-.450*** | .156 | -.198 | -.348 | -.291 |
| (3) | .125 | .059 | -.022 | -.118 | -.061 | .006 | .253 | .394 | .001 | .123 | .258 | -.378 | .273 | .142 | .077 |
| (4) | .250 | -.008 | -.181 | .137 | -.096 | .020 | .225 | .288 | -.244 | .047 | .236 | -.165 | .255 | .300 | .245 |
| (5) | .201 | .031 | -.106 | .001 | -.085 | .014 | .263 | .378 | -.124 | .096 | .273 | -.306 | .289 | .240 | .171 |
| (6) | .101 | .180 | -.125 | .255 | .003 | .216 | .198 | .177 | .355 | .086 | .353 | -.291 | .260 | .281 | .344 |
| (7) | -.161 | .021 | -.031 | -.038 | .101 | -.145 | .180 | -.055 | .319 | -.025 | .380 | -.288 | .270 | .302 | .276 |
| (8) | -.066 | .131 | .139 | .155 | .068 | .065 | .134 | .035 | .203 | .110 | **.467*** | -.220 | .132 | .152 | .254 |
| (9) | -.132 | -.036 | .129 | -.009 | .179 | -.105 | -.050 | .064 | -.001 | .191 | .390 | -.300 | .002 | -.080 | .041 |
| (10) | -.353 | -.127 | .045 | **-.449*** | .056 | -.288 | -.138 | -.108 | -.130 | **-.491*** | .145 | **-.484*** | -.114 | -.057 | -.129 |
| (11) | **-.413*** | -.221 | -.046 | -.292 | .376 | .232 | .052 | .091 | -.324 | -.282 | -.114 | -.328 | -.263 | -.224 | **-.442*** |
| (12) | .260 | .268 | .290 | .252 | -.311 | .115 | .118 | .322 | .280 | .397^*^ | **.602**** | -.026 | **.510*** | .316 | .360 |
| (13) | -.064 | .082 | .179 | .025 | -.038 | .014 | .034 | .164 | .102 | .142 | **.547**** | -.144 | .424 | .213 | .283 |
| (14) | -.184 | -.146 | -.328 | .027 | .148 | -.212 | -.393 | .049 | -.040 | -.235 | -.091 | .105 | .152 | .061 | -.115 |
| Mild Cognitive Impaired group | | | | | | | | | | | | | | | |
| (1) | .174 | -.015 | .343 | .375 | -.161 | **.547*** | **.533*** | .221 | .064 | .044 | .080 | .051 | **.578**** | .435 | -.016 |
| (2) | -.413 | .095 | -.135 | **-.599**** | .313 | -.034 | **-.631**** | -.373 | .026 | -.261 | -.537^*^ | -.094 | **-.497*** | -.496 | -.160 |
| (3) | **.670**** | .138 | .083 | .122 | -.124 | -.549 | .115 | .134 | -.050 | -.221 | .283 | .069 | .307 | .209 | -.049 |
| (4) | **.491*** | .075 | -.046 | .067 | .050 | -.229 | .014 | .095 | .007 | -.264 | .191 | .087 | .355 | .324 | -.101 |
| (5) | **.616**** | .113 | .022 | .100 | -.040 | -.438 | .069 | .120 | -.023 | -.255 | .250 | .082 | .348 | .277 | -.078 |
| (6) | **.470*** | .254 | .187 | .297 | .130 | -.378 | .094 | .397 | .249 | .065 | .084 | -.040 | .321 | .520 | .031 |
| (7) | .268 | .412 | .083 | .089 | .182 | -.439 | -.127 | .138 | .220 | -.193 | -.006 | -.019 | .203 | .307 | -.007 |
| (8) | -.014 | .266 | -.137 | **.589**** | .038 | .058 | **.436*** | **.614**** | .225 | .350 | **.449*** | -.021 | .139 | .424 | **.542*** |
| (9) | -.081 | .211 | -.266 | .402 | .221 | -.161 | .171 | .342 | .131 | .111 | .193 | -.082 | .166 | .307 | **.546*** |
| (10) | -.061 | -.201 | **-.706**** | -.067 | .273 | **-.660*** | -.101 | -.015 | -.132 | -.125 | .018 | -.083 | -.149 | -.115 | .147 |
| (11) | .125 | .229 | -.301 | .062 | .102 | -.465 | .116 | .206 | -.177 | .113 | -.061 | -.213 | -.055 | .010 | .178 |
| (12) | .343 | -.003 | -.256 | -.157 | -.197 | **-.751**** | -.098 | .089 | -.160 | .062 | .105 | -.025 | -.030 | -.287 | -.119 |
| (13) | .373 | .104 | -.246 | -.111 | -.213 | **-.812**** | -.071 | .090 | -.147 | .049 | .184 | -.063 | -.044 | -.268 | -.083 |
| (14) | .493 | -.238 | **-.634*** | -.076 | .116 | **-.598*** | .258 | .066 | **-.633*** | .012 | -.055 | -.389 | .207 | .085 | .006 |

Legend: (1) % of Normal-appearing white matter volume in ICV, (2) % of CSF volume in ICV, (3) % Left hippocampal volume in ICV, (4) % Right hippocampal volume in ICV, (5) % Total hippocampal volume in ICV, (6) % Left caudate nucleus grey matter volume in ICV, (7) % Right caudate nucleus grey matter volume in ICV, (8) % Left putamen grey matter volume in ICV, (9) % Right putamen grey matter volume in ICV, (10) % Left globus pallidus grey matter volume in ICV, (11) % Right globus pallidus grey matter volume in ICV, (12) % Left thalamus grey matter volume in ICV, (13) % Right thalamus grey matter volume in ICV, (14) % parahippocampal volume in ICV, VSTM: Visual short term memory, ACE: Adenbrook’s Cognitive Examination, TMT: Trail Making Task, HVLT: Hopkins Verbal Learning Task

Table S43. Bivariate Pearson's (r) correlations between imaging and cognitive variables in the whole sample (i.e. scores from the cognitive tests)

|  | (A) | (B) | (C) | (D) | (E) | (F) | (G) | (H) | (I) | (J) | (K) | (L) | (M) | (N) | (O) |
| --- | --- | --- | --- | --- | --- | --- | --- | --- | --- | --- | --- | --- | --- | --- | --- |
| (1) | 0.139 | 0.099 | **0.320* | 0.095 | -0.274 | 0.27 | **0.305* | 0.124 | 0.074 | -0.003 | 0.27 | -0.028 | 0.237 | **0.337* | 0.08 |
| (2) | -0.257 | -0.114 | -0.179 | **-0.309* | 0.281 | -0.063 | **-0.341* | -0.262 | -0.076 | -0.134 | ***-0.487* | 0.097 | -0.274 | ***-0.414* | -0.284 |
| (3) | ***0.428* | 0.196 | 0.153 | 0.175 | -0.149 | 0.054 | 0.274 | **0.367* | 0.106 | 0.134 | **0.347* | -0.211 | 0.257 | **0.367* | 0.126 |
| (4) | ***0.441* | 0.181 | 0.088 | **0.304* | -0.125 | 0.197 | 0.261 | **0.356* | 0.098 | 0.171 | **0.353* | -0.063 | **0.367* | ***0.450* | 0.222 |
| (5) | ***0.465* | 0.202 | 0.13 | 0.254 | -0.147 | 0.133 | 0.286 | ***0.386* | 0.109 | 0.163 | **0.374* | -0.148 | **0.332* | ***0.436* | 0.185 |
| (6) | 0.297 | 0.246 | 0.079 | 0.29 | 0.025 | 0.081 | 0.184 | 0.289 | **0.318* | 0.136 | 0.268 | -0.193 | -0.248 | **0.365* | 0.224 |
| (7) | 0.172 | **0.332* | 0.171 | 0.222 | 0.045 | 0.029 | 0.135 | 0.198 | **0.361* | 0.118 | **0.297* | -0.144 | 0.268 | **0.375* | 0.227 |
| (8) | 0.009 | 0.234 | 0.082 | ***0.412* | 0.003 | 0.13 | **0.320* | **0.323* | 0.275 | **0.295* | ***0.482* | -0.149 | 0.157 | 0.212 | ***0.421* |
| (9) | -0.049 | 0.13 | 0.023 | 0.25 | 0.149 | 0.026 | 0.104 | 0.222 | 0.124 | 0.205 | **0.341* | -0.222 | 0.087 | 0.146 | **0.298* |
| (10) | -0.109 | -0.032 | -0.164 | 0.032 | 0.048 | -0.113 | 0.038 | 0.123 | 0.053 | 0.02 | 0.238 | **-0.315* | -0.097 | -0.045 | 0.141 |
| (11) | -0.093 | 0.074 | 0 | 0.132 | 0.163 | 0.136 | 0.215 | **0.293* | -0.036 | 0.169 | 0.093 | -0.274 | -0.057 | -0.029 | -0.023 |
| (12) | 0.257 | 0.104 | 0.019 | -0.027 | -0.231 | -0.076 | -0.015 | 0.147 | 0.013 | 0.111 | **0.313* | -0.025 | 0.122 | 0.091 | 0.091 |
| (13) | 0.125 | 0.063 | -0.032 | -0.077 | -0.1 | -0.138 | -0.04 | 0.078 | -0.057 | 0.029 | **0.301* | -0.105 | 0.063 | 0.082 | 0.063 |
| (14) | 0.083 | 0.007 | -0.148 | 0.223 | 0.008 | -0.071 | -0.017 | 0.223 | 0.083 | 0.109 | 0.116 | 0.086 | **0.310* | 0.202 | 0.088 |

Legend: (1) % of Normal-appearing white matter volume in ICV, (2) % of CSF volume in ICV, (3) % Left hippocampal volume in ICV, (4) % Right hippocampal volume in ICV, (5) % Total hippocampal volume in ICV, (6) % Left caudate nucleus grey matter volume in ICV, (7) % Right caudate nucleus grey matter volume in ICV, (8) % Left putamen grey matter volume in ICV, (9) % Right putamen grey matter volume in ICV, (10) % Left globus pallidus grey matter volume in ICV, (11) % Right globus pallidus grey matter volume in ICV, (12) % Left thalamus grey matter volume in ICV, (13) % Right thalamus grey matter volume in ICV, (14) % parahippocampal volume in ICV, (A) Short term memory shape-only with 3 objects, (B) Short term memory shape-colour binding with 3 objects, (C) Short term memory shape-colour binding with 2 objects, (D) Addenbrooke’s Cognitive Examination, (E) Trail Making Task B-A, (F) Hopkins Verbal Learning Task recognition, (G) Hopkins Verbal Learning Task delayed recall, (H) Hopkins Verbal Learning Task total recall, (I) Total FAS, (J) Animal fluency, (K) Digit symbol, (L) Rey figure copy, (M) Rey figure immediate recall, (N) Rey figure delayed recall, (O) Graded naming test.
